# Determinants of Indoor NO_2_ and PM_2.5_ Concentration in Senior Housing with Gas Stoves

**DOI:** 10.3390/toxics12120901

**Published:** 2024-12-11

**Authors:** Khafayat Kadiri, David Turcotte, Rebecca Gore, Anila Bello, Susan R. Woskie

**Affiliations:** 1 Department of Public Health, University of Massachusetts Lowell, Lowell, MA 01854, USA; anila_bello@uml.edu (A.B.); susan_woskie@uml.edu (S.R.W.); 2 Center for Community Research and Engagement, University of Massachusetts Lowell, Lowell, MA 01854, USA; david_turcotte@uml.edu; 3 Department of Economics, University of Massachusetts Lowell, Lowell, MA 01854, USA; 4 Department of Biomedical Engineering, University of Massachusetts Lowell, Lowell, MA 01854, USA; rebecca_gore@uml.edu

**Keywords:** nitrogen dioxide (NO_2_), particulate matter (PM), gas stoves

## Abstract

Nitrogen dioxide (NO_2_) and particulate matter of 2.5 microns (PM_2.5_) are air pollutants that impact health, especially among vulnerable populations with respiratory disease. This study identifies factors influencing indoor NO_2_ and PM_2.5_ in low-income households of older adults with asthma who use gas stoves in Lowell, Massachusetts. Environmental sampling was conducted in 73 homes, measuring NO_2_, PM_2.5_, fractional stove-use, temperature, and humidity for 5–7 days. Participants were recruited between December 2020 and July 2022. Questionnaires were used to collect data on factors influencing indoor NO_2_ and PM_2.5_ concentrations. Daily outdoor NO_2_ and PM_2.5_ concentrations were obtained from a United States Environmental Protection Agency (EPA) monitoring station. Paired *t*-tests were conducted between indoor and outdoor NO_2_ and PM_2.5_ concentrations, and linear regression was used to evaluate factors influencing indoor NO_2_ and PM_2.5_ concentrations. The average indoor concentration for NO_2_ and PM_2.5_ were 21.8 (GSD = 2.1) ppb and 16.2 (GSD = 2.7) µg/m^3^, respectively. Indoor NO_2_ and PM_2.5_ concentrations exceeded outdoor concentrations significantly. In multiple regression models, season and pilot light stove use significantly predicted indoor NO_2_. Season and air freshener use for 6–7 days/week significantly predicted indoor PM_2.5_. Season-influenced higher indoor concentrations are likely due to reduced ventilation in colder months in the Northeast U.S.

## 1. Introduction

Indoor air quality (IAQ) has proven to be a crucial factor in the well-being of individuals. There has been substantial evidence of poor IAQ’s adverse effects on health and quality of life [1,2,3,4]. Indoor air pollution has been associated with respiratory illness, poor lung development, and mortality [5]. IAQ is especially important for the health of older adults who spend about 90% of their time indoors [6]. More than half of the human body’s daily air intake is inhaled in the home, and most illnesses related to environmental exposures occur from indoor air exposures [7]. IAQ is a complex function of outdoor and indoor sources of pollution, environmental conditions, housing characteristics, and behavioral factors. Potential factors that influence IAQ include physical and chemical properties of indoor pollutants, the use of household products, appliances, combustion sources (such as burning fuels, coal, and wood; tobacco products; and candles), building and furnishing materials, building characteristics (infiltration and ventilation rates), occupant behavior and activities (opening of windows, tobacco smoking, cleaning, cooking, and use of extractor fans), heating and cooling systems, humidification devices, moisture processes, electronic equipment, products for household cleaning, and pets [8]. According to the United States Environmental Protection Agency (EPA), indoor levels of pollutants may be 100 times higher than outdoor pollutant levels and have been ranked among the top 5 environmental risks to the public [3].

### 1.1. Nitrogen Dioxide

Nitrogen dioxide (NO_2_) is a by-product of combustion produced by motor vehicles, energy generation, and other outdoor sources involving combustion, as well as indoor sources such as gas appliances and kerosene heaters [3,8,9,10]. Nitric oxide and nitrogen dioxide are considered the most important of the oxides of nitrogen (NO_x_) in terms of public health concern [11]. Nitrogen oxides are irritant gases that can cause bronchoconstriction, airway hyper-responsiveness, and airway inflammation, with an increased risk of asthma exacerbations, bronchitis, and wheezing [12]. Exposure to elevated indoor NO_2_ concentrations can lead to symptoms in individuals with asthma, including declining lung function, chest tightness, shortness of breath, wheezing, cough, nocturnal symptoms, increased number of asthma attacks, increased inhaler use, and decreased forced expiratory volume (FEV1) [8,13,14,15,16,17]. Higher indoor NO_2_ concentrations have been associated with reduced Asthma Control Test (ACT) scores in adults with asthma [18]. A review by Hesterberg et al. found that the populations most susceptible to NO_2_-induced pulmonary effects were individuals with asthma or allergic diseases [19].

### 1.2. Particulate Matter

Particulate matter (PM) is a complex mixture of organic and inorganic chemicals suspended in the atmosphere, which vary by geographic location. Indoor particles are ambient particles of different size fractions (typically reported as PM_10_, and PM_2.5_) that either infiltrate from outdoors or are generated by indoor sources. Some studies have shown that PM concentrations are higher indoors than outdoors due to generation in the indoor environment [20,21]. Indoor PM sources include smoking, cooking (mainly using kerosene and biomass fuels), wood stoves, furnaces, use of incense and candles, cleaning, the presence of pets, and human activities [8,20,22]. High indoor PM_2.5_ concentrations directly impact human health [23,24]. PM exposures have been linked to a decline in pulmonary function and cardiovascular events through inflammation. In this study, we focus on PM_2.5_, because it is smaller in size and penetrates deeper into the respiratory system, where it can cause inflammation, and exacerbate asthma [25,26]. The inhalation of PM_2.5_ can irritate the airways and trigger inflammatory responses in the respiratory system, worsening existing asthma conditions [8,23,27]. Short-term exposure to PM_2.5_ has also been associated with an increased risk of hospital admissions and emergency room visits due to severe asthma attacks [16]. Long-term exposure to PM_2.5_ has been associated with asthma development in children and adults and lung function decline [16].

### 1.3. Gas Stoves’ Impact on Indoor NO_2_ and PM_2.5_

Gas stoves primarily utilize methane or propane as their carbon sources. The combustion of these gases produces pollutants, including NO_2_ and PM_2.5_ [12]. Gas stoves may pose a greater health risk due to their proximity to individuals and their children and their potential to produce concentrated emissions during cooking activities [28,29,30]. Indoor combustion from gas stoves has been identified as an important source of indoor NO_2_ [31,32,33]. During cooking, peaks in NO_2_ are generated that may reach hundreds of parts per billion (ppb), exceeding 100 ppb—the outdoor short-term 1 h National Ambient Air Quality Standard (NAAQS)—after only a few minutes [28,29,30]. Personal exposures to NO_2_ are higher for persons living in homes with gas stoves than those living in homes with electric stoves [34]. Short- and long-term exposure to NO_2_ has been shown to have many adverse health effects. Belanger et al. conducted a sensitivity analysis that showed an increased risk of asthma attacks in children with short-term exposure to NO_2_: for every 5 ppb increase in NO_2_, above a threshold of 6 ppb, there was an increase in the risk of wheezing and the need for medication [19]. Factors affecting indoor NO_2_ and PM_2.5_ concentrations from gas stoves include usage frequency, maintenance, type, cookware, gas used, number of appliances, burner adjustment, and use of exhaust fans [35]. Homes missing exhaust hoods have been observed to generate NO_2_ levels above the outdoor standard [36]. Numerous activities of residents may generate and resuspend PM, increasing the indoor PM concentration [27].

Understanding the relationships and the influence of various indoor factors on indoor air quality is crucial for prioritizing control measures and mitigation action plans [37]. The main objective of this study was to determine which factors are most important in predicting indoor concentrations of NO_2_ and PM_2.5_ in the low-income residential apartments of older adults with asthma. This is vital because it focuses on air pollution risks in senior housing, where residents are especially vulnerable to indoor exposure to NO_2_ and PM_2.5_ from gas stoves. The findings will contribute to strategies to improve air quality, such as better ventilation, transitioning to electric stoves, and informing policies to protect seniors’ health.

## 2. Materials and Methods

The data used in this study were obtained from 73 homes as part of an intervention study to reduce indoor NO_2_ and PM_2.5_ and other environmental triggers in the homes of older adults living in low-income apartments in Lowell, Massachusetts. Study participants were older adults with self-reported doctor-diagnosed asthma, living in low-income housing, using a gas stove, and who experienced no prior intervention to reduce PM_2.5_ and NO_2_. Participants were recruited through community outreach by researchers at the University of Massachusetts Lowell and Community Health Workers (CHWs) from the Lowell Community Health Center, and Lowell Housing Authority (LHA). The study materials were distributed in residential apartments across Lowell via a CHW contact database. The CHWs contacted potential participants, verified their eligibility for the study, and enrolled participants. Eligible participants provided verbal informed consent before participating in the study, as we changed from written informed consent due to health risk to participants from COVID-19. Individuals with life-threatening asthma, history of respiratory failure, mental incoherency, current residents of a long-term care facility, and/or participating in other interventional studies were excluded. Data collected were stored in the Research Electronic Data Capture (REDCap), an online application system hosted at the University of Massachusetts Chan Medical School. REDCap is a secure, web-based software platform designed to support data capture for research surveys [38,39].

### 2.1. Environmental Sampling

Environmental sampling was conducted in each participant’s home for 5–7 days to measure NO_2_, PM, fractional stove use, temperature, and humidity simultaneously. Environmental sampling was conducted between December 2020 and July 2022. Our sampling strategy focused on short-term exposure measurements, with a sampling duration of 5–7 days, relevant for investigating the impact of acute exposures on the aggravation of asthma symptoms among the participants [40,41].

NO_2_ sampling was conducted using a passive NO_2_ Ogawa sampler (Ogawa & Co., USA Inc., Pompano Beach, FL, USA) that contained a coated filter housed between two screens inside a plastic chamber [42]. The assembled sampler was stored in the refrigerator inside a resealable plastic bag enclosed inside an opaque plastic container before and after sampling. The samplers were transported to the field and back inside the plastic containers. The NO_2_ sampler was placed next to a TRIX−8 multi-use temperature logger (manufactured by Logtag North America Inc., Lafayette, NJ, USA [43]), which measured temperature and humidity during the sampling period. The Logtag was configured to record every 5 min. Data from the temperature and humidity logger was downloaded, and the average temperature and humidity for the sampling period were recorded in REDCap. NO_2_ samples were shipped for analysis by colorimetry to RTI International, Durham (NC, USA). Laboratory results consisted of blank adjusted NO_2_ concentration in ppb using the average indoor temperature and humidity data collected during the sampling period.

PM sampling was conducted with a direct reading optical particle counter, the Dylos 1700-PM (Dylos Corporation, Riverside, CA, USA), which simultaneously measured mass concentration in two size fractions (PM_2.5_ and PM_10_, reported in micrograms per cubic meter (µg/m^3^)) and numerical concentration in two size fractions (>0.5 mm and >2.5 mm per 0.01 cubic feet of air). The instrument measured the average PM for each minute [44,45,46]. Dylos 1700 is a low-cost particle counter that has been successfully used to measure indoor particles providing low noise levels that minimize the burden on study participants [47,48]. Data were downloaded with the Dylos logger software version 3.0, and the average and maximum PM_2.5_ concentrations were recorded (in this study, we focused only on PM_2.5_). The Dylos monitor, NO_2_ sampler, and temperature and humidity logger were placed in the main living area on a table. The sampling was conducted in the living room due to the proximity to the gas stoves and the kitchens being extremely small and lacking adequate space for sampling devices [49,50].

Gas stove use was measured with an iButton semiconductor temperature sensor (DS1921G-F5, Analog Devices, Inc., Wilmington, MA, USA). This device has been used previously in studies of in-home biomass stoves in low-income countries [51]. An iButton unit was placed between the burners on each stove side. The two iButtons were layered with silicon sheets and a bottle cap over them to reduce damage from spillage during cooking and extreme temperatures, and the iButtons were secured to the stove top using fire-resistant tape. The iButtons measured the temperature on the stovetop every 6 min during the sampling period. The data were downloaded, and the fractional stove use was estimated for the sampling period and recorded in REDCap. Stove use was determined as the fraction of time during which the temperature on the stove’s surface was above the cutpoint temperature during sampling in each home. The temperature cutpoints used to determine stove use were as follows: (a) if the Logtag average temperature was less than 24 °C, then the cutpoint was 24 °C or above; (b) if the Logtag average temperature was over 24 °C, then the cutpoint was the Logtag average temperature plus 2 °C; and (c) if the stove had a pilot light, the modal stove temperature from the iButton plus 2 °C (we considered this only for pilot stoves because they have a small flame that burns continuously, even when the burners are unused, making the surface constantly warm).

Other potential factors influencing NO_2_ and PM_2.5_: Data on each predictor were obtained through a survey administered by the study team at the time of sampling. Data were collected on stove type; room type; outdoor tobacco use; presence of a range hood; dryer use; HEPA vacuum use; nearby trucks; a gas station close to residence; indoor activities such as air freshener, incense, and candle use; and home characteristics such as floor type or presence of pests, mold, and potential factors that could influence NO_2_ and PM_2.5_ concentrations. In addition to indoor monitoring, we obtained data on outdoor NO_2_ and PM_2.5_ levels from the closest EPA monitoring station in Chelmsford, MA. Due to the low variability in outdoor NO_2_ and PM_2.5_ levels during the week, their concentrations on the first day of the sampling period were compared with the indoor average concentrations for the sampling period.

### 2.2. Statistical Analysis

Descriptive statistics were performed to characterize indoor concentrations of NO_2_ and PM_2.5_ at the study sites. The NO_2_ and PM_2.5_ concentrations were log-transformed using natural logarithms (ln) to meet the distributional normality assumption. We conducted simple regression analyses to identify factors that independently predicted indoor NO_2_ and PM_2.5_ concentrations using the following variables: stove usage, outdoor NO_2_ and PM_2.5_, sampling season, indoor temperature, indoor humidity, stove type, room type, outdoor tobacco use, presence of range hood, HEPA vacuum use, nearby trucks, gas station close to residence, and air freshener use. Predictor variables that were statistically significant in predicting indoor NO_2_ and PM_2.5_ in simple linear regression analysis were included in a multiple regression analysis. Fractional stove usage was always included in the multiple predictor regression models as our primary hypothesis was that stove usage was a critical factor in indoor concentrations. A paired *t*-test on the ln-transformed concentrations was conducted to identify if there was a difference between outdoor and indoor NO_2_ and PM_2.5_ during the sampling period. All statistical analyses were performed on Statistical Analysis System) (SAS) version 9.4.

## 3. Results

### 3.1. Characteristics of Participants and Homes

**Figure 1:**
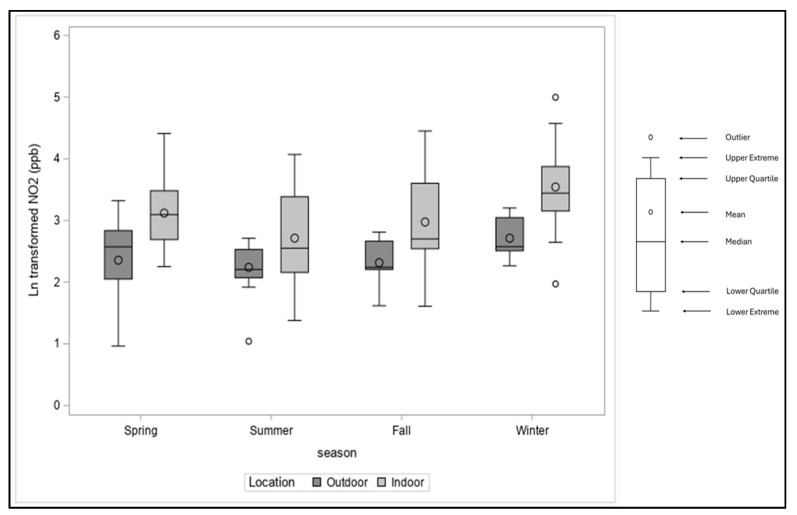
Box plots of indoor and outdoor ln-transformed NO_2_ levels by season.

**Figure 2:**
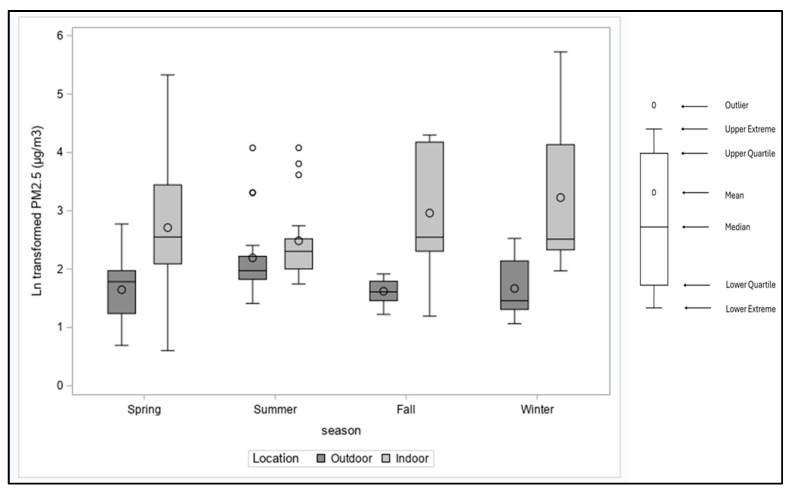
Box plots of indoor and outdoor ln-transformed PM_2.5_ levels by season.

Eighty-one older adults from 77 homes were enrolled in the study: 28% identified as male, 38% White, 17% Asian, 2% Black or African American, 1% American Indian/Alaska Native, and 41% unknown race, while 44% identified as Hispanic. Housing data were collected from 73 homes; the housing units in the study were single-family attached row houses that shared adjacent walls (27%) and apartments in multi-family unit buildings (73%). Housing characteristics are listed in Table 1. We evaluated the stove usage in these homes and observed an average of 0.3 (30%) fractional stove use in these homes with a range of 0–0.8. The seasonal variations in NO_2_ and PM_2.5_ concentration in indoor air are presented in Figure 1 and Figure 2.

### 3.2. Indoor and Outdoor NO_2_ and PM_2.5_ Concentrations

Environmental sampling was conducted in all seasons: 44% in the Spring, 26% in the Summer, 19% in the Winter, and 11% in the Fall. During the sampling period, the average indoor concentration of NO_2_ was 21.8 ± 2.1 ppb, while the outdoor concentration of NO_2_ was 10.9 ± 1.7 ppb. A paired *t*-test on the natural log (ln) concentrations showed a statistically significant difference between indoor and outdoor NO_2_ (Table 2), with average indoor NO_2_ higher than outdoor concentrations across all seasons (Figure 1). The indoor concentration of PM_2.5_ was 16.2 ± 2.8 µg/m^3^, while the outdoor concentration of PM_2.5_ during the sampling period was 5.9 ± 1.8 µg/m^3^. A paired *t*-test on the ln concentrations showed a statistically significant difference between indoor and outdoor PM_2.5_ (Table 2), with average indoor PM_2.5_ higher than outdoor concentrations across all seasons except summer (Figure 2).

### 3.3. Predictors of Indoor NO_2_ and PM_2.5_

Outdoor NO_2_ and PM_2.5_ concentrations: We observed that the average outdoor NO_2_ concentration on day 1 of the sampling period did not predict the indoor concentration (*p*-value = 0.33, Table 3), and outdoor PM_2.5_ concentrations on day 1 of sampling did not predict indoor PM_2.5_ concentrations (*p*-value = 0.51, Table 4). Stove use: The fractional stove usage did not significantly predict indoor NO_2_ (*p*-value = 0.65, Table 3). However, stove type was a significant predictor of indoor NO_2_ concentrations, with increased concentration in homes with pilot lights on their gas stove (*p*-value = 0.04, Table 3). Stove use did not significantly predict indoor PM_2.5_ levels (*p*-value = 0.71, Table 4) nor did stove type (*p*-value = 0.3, Table 4).

Season: Season was a significant predictor of indoor NO_2_ concentration, with lower concentrations in the Summer, Fall, and Spring compared to Winter (*p*-values: Fall = 0.05; Spring = 0.05; Summer = 0.0006, Table 3). The Summer season had a significantly lower concentration of indoor PM_2.5_ when compared to the Winter season (*p*-value = 0.03, Table 4). However, there was no significant impact during the Fall and Spring seasons (*p*-values: Fall = 0.52; Spring = 0.1, Table 4). Housing characteristics: Using air fresheners for more than six days a week and living near a gas station (within 0.5 miles) significantly increased the indoor concentration of PM_2.5_ (*p*-values: air fresheners = 0.0016; gas station = 0.01, Table 4), but did not significantly predict indoor NO_2_ levels.

In the multiple regression model of ln NO_2_, we included stove usage, season, and stove type. We built these models by including an a priori variable for stove use and the variables significantly impacting ln NO_2_ and ln PM_2.5_ from the single-predictor models. The results showed that stove usage did not predict indoor NO_2_ (*p*-value = 0.47, Table 5) while stove type (*p*-value = 0.0004, Table 5) and season (*p*-values: Fall = 0.01; Spring = 0.01; and Summer < 0.0001, Table 5) remained significant predictors of indoor NO_2_. As with the single-predictor models, the lowest concentrations were in the summer, followed by fall and spring, compared to winter (Table 5).

In the multiple-predictor regression model of ln PM_2.5_, we included stove usage, season, and air freshener use. The results showed that stove usage did not predict indoor PM_2.5_ (*p*-value = 0.76, Table 6), while air freshener use for 6–7 days a week (*p*-value = 0.001) and season (*p*-values: Spring = 0.05; Summer = 0.04, Table 6) remained significant predictors of ln PM_2.5._ As with the single-predictor models, the lowest concentrations were in the summer, followed by spring and fall, compared to winter (Table 6).

## 4. Discussion

In this study, we report indoor concentrations of NO_2_ and PM_2.5_ in low-income housing units using gas stoves at levels significantly higher than outdoor concentrations across seasons. Earlier studies have reported that outdoor PM_2.5_ and NO_2_ are significant sources of indoor PM_2.5_ and NO_2_ when the outdoor concentrations are higher than indoor concentrations [9,23,24,27,32]. However, in this study, indoor PM_2.5_ and NO_2_ concentrations were higher than outdoor concentrations, and outdoor levels did not predict indoor concentrations in univariate models (Table 3 and Table 4). These results indicate the importance of identifying indoor sources for indoor NO_2_ and PM_2.5_ levels.

The maximum NO_2_ indoor concentration measured was 148 ppb, which is higher than the annual U.S. EPA outdoor standards of 53 ppb (annual) and 100 ppb (1 h daily maximum) [6,52]. These results indicate an increased risk for adverse respiratory conditions among more susceptible individuals. While the average indoor NO_2_ measured in our study was 28.6 ppb and the Geometric Mean (~median) was 21.8 ppb, children exposed to levels of 25.9 ppb have been reported to have an increased likelihood of wheezing, shortness of breath, and chest tightness [13]. Acute short-term exposure to NO_2_ from single episodes of gas cooking has been related to immediate airflow limitation [53]. Continued exposure from repeated episodes of gas cooking in asthmatic women has been associated with greater use of rescue bronchodilators [53]. A longitudinal study by Hansel et al. found a significant association between NO_2_ concentrations and quality of life, the study found that there was a relationship between indoor NO_2_ concentrations in the main living area and increased dyspnea and higher rescue medication use, while bedroom NO_2_ concentrations were associated with a higher risk of nocturnal symptoms and severe COPD exacerbations [54].

The average indoor concentration for PM_2.5_ was 30.4 µg/m^3^, which exceeded the annual and 24 h World Health Organization (WHO)-recommended level of 5 µg/m^3^ and 15 µg/m^3^, respectively, and the U.S. EPA annual recommendation of 9–10 µg/m^3^ [6,52]. This indicates that the indoor levels are at levels that are harmful to the well-being of individuals. The outdoor concentrations were much lower (average 7.3 µg/m^3^). The high weekly indoor concentrations we observed are concerning, given the evidence of potential adverse health impacts. Hansel et al. found that a 10 μg/m^3^ increase in PM_2.5_ concentrations was associated with 44% higher odds of nocturnal symptoms and 50% higher odds of severe COPD exacerbation [54].

### 4.1. Stove Usage Impact on Indoor NO_2_ and PM_2.5_

Using gas stoves has been observed to contribute to indoor concentrations of NO_2_ [2,8], and cooking duration has also been shown to increase indoor NO_2_ concentrations [9,30,53,55]. However, in this study, stove use did not influence indoor concentrations of NO_2_. Other studies have evaluated cooking habits that have contributed to indoor NO_2_ concentrations, such as cooking methods and duration of range hood use while cooking [56]. Ng et al. observed the highest exposure of indoor NO_2_ in the first 5–10 min of cooking and reduced exponentially, suggesting that exposure to NO_2_ is highest during the start-up of the gas appliances and in the initial 10 min of gas cooking when fuel combustion is still incomplete [53]. Our results were based on average concentrations for 5–7 sampling days. Also, Ng et al. observed reduced levels of NO_2_ when combustion was completed with increased ventilation. In this study, 47% of the participants reported having a recirculating stove ventilation hood, but we did not collect data on when/how often the stove ventilation was used. Our data collection method explains why we did not observe stove usage as a predictor of indoor NO_2_ because we measured stove usage and NO_2_ as an average of the sampling period rather than direct measurement during cooking [53]. We observed increased indoor NO_2_ when the homes had gas stoves with a pilot light (*n* = 5), which has also been observed in prior studies [57,58,59]. A pilot light is a small flame that burns continuously on a gas stove, even when the burners are unused. Since 1990, gas stoves sold in the United States have been required to have electronic ignition systems, whereas previously, many had continuously burning pilot lights, which is a steady source of emissions [60]. Similar to NO_2_, gas stove usage did not predict indoor concentrations of PM_2.5._ These results are similar to Zusman et al., which showed that cooking-related variables did not predict indoor PM_2.5_ [33]. However, other studies have linked indoor PM_2.5_ concentrations to the presence of gas stoves [8,20,55] and reported that household cooking habits contribute to PM_2.5_ concentrations [56,61]. We did not focus on cooking patterns/methods that could potentially impact short-term emissions. Also, our sample size was relatively small, potentially reducing statistical power. These factors may explain why we did not observe stove usage as a predictor of indoor PM_2.5_.

### 4.2. Seasonal Variation in NO_2_ and PM_2.5_

The highest indoor concentrations of NO_2_ and PM_2.5_ were observed in the winter (Figure 1 and Figure 2), which is similar to earlier studies that observed increased indoor concentrations of NO_2_ and PM_2.5_ during months with higher indoor heating (winter) and in homes with gas stoves [62,63,64,65,66]. Although the average stove use was highest during the spring season, we speculated that the increase in NO_2_ observed in the winter season may most likely be attributable to reduced ventilation during the winter season which has been observed in similar studies [62,65]. Some studies observed an increased concentration of indoor NO_2_ in the summer due to infiltration from outdoors when windows are open [67]; however, we observed reduced indoor and outdoor NO_2_ and PM_2.5_ concentrations in our study in the summer. Seasonal variations have also been observed in other studies [68]. However, we did not collect data on the ventilation practices in the homes or conduct direct paired outdoor monitoring. Instead, we relied on outdoor concentrations within the study region from the EPA, making it challenging to explain the seasonal influence of outdoor concentrations on indoor NO_2_ and PM_2.5_.

### 4.3. Other Factors That Influence Indoor NO_2_ and PM_2.5_

Air Freshener Use—Using air fresheners for 6–7 days a week significantly increased indoor concentrations of PM_2.5_, similar to studies that found that air freshener usage contributed to asthma exacerbations and indoor PM_2.5_ concentrations [3,27]. While information on the frequency of air freshener use was collected, the type of air fresheners used in these homes were not collected. Park et al. found that using air freshener sprays did not increase PM_2.5_ above the WHO standard of 15 µg/m^3^ [52], but using scented candles increased PM_2.5_ [27]. An exposure and health risk analysis found that using air freshener sprays and active diffusers contributes to elevated levels of PM_2.5_, exceeding WHO standards [69]. Li et al. found that using air fresheners was significantly and negatively associated with Forced vital Capacity (FVC) in children [70]. Air fresheners have been observed to be suspended in the air, and air fresheners produce particulate matter through the emission of VOCs and other chemicals that can undergo chemical reactions and physical transformations in the air, leading to the formation of particles of various sizes [27,71]. In this study, we did not include air fresheners in the multiple predictor model for NO_2_ because it was not significant in the single predictor model and earlier studies did not observe air fresheners emitting NO_2_ [72,73,74,75,76]. Evaluating the type of air fresheners used in homes will be important in future studies to understand PM_2.5_ levels indoors.

Presence of gas station—A gas station within ½ mile of the participant’s home was found to increase indoor PM_2.5_ concentrations significantly. The presence of a gas station directly infers that many vehicles pass through, making it a high-traffic area. Earlier studies have observed that high traffic contributes to indoor PM_2.5_ concentrations due to car emissions [21,33]. PM_2.5_ from vehicular emissions is also influenced by wind speed and direction [77]. We excluded the presence of gas stations in the multiple regression model due to the small sample size; with a small sample size, the estimates of regression coefficients can be biased and have high variance.

Outdoor Tobacco Use—Tobacco smoke residues can linger and become a source of long-term exposure to harmful pollutants. PM_2.5_ can remain airborne or be absorbed into indoor surfaces and dust particles and stay for many hours after smoking has ended [78]. Fifty-one percent of our participating homes reported tobacco smoke entering their apartment despite these homes being smoke-free. Indoor locations where smoking is banned are not entirely free from environmental tobacco smoke (ETS). When smoking is allowed outdoors near entrances, it can drift indoors to adjacent apartments [79]. Nevertheless, unlike other studies that found outdoor tobacco to be a contributor to indoor PM_2.5_ [33,56], this study did not observe outdoor tobacco use to contribute to indoor PM_2.5_ (Table 3).

Use of Range hood—A stove hood/mechanical ventilation in the kitchen was not observed to reduce the indoor concentration of NO_2_ and PM_2.5_. Forty-seven percent of the participating homes reported having a recirculating vent in the kitchen over the stove that did not vent outside. However, other studies have found that using an exhaust hood that vents outdoors can reduce indoor NO_2_ and PM_2.5_ concentrations [24,33,80]. Also, the lack of reduction in NO_2_ and PM_2.5_ with the presence of a stove ventilation hood may be due to the lack of use of a ventilation hood while cooking, recirculation mechanism, and variability in cooking habits, which have been observed to contribute to indoor concentrations of PM_2.5_ and NO_2_ [56]. Mullen et al. observed that the effectiveness of stove hoods in homes was reduced by the fact that 35% of participants (among those having ventilation hoods) reported using it on medium or low speed, and 70% of participants reported cooking primarily on the front burners in their air quality study [81].

### 4.4. Potential Interventions

Several studies have identified ways to reduce indoor NO_2_ and PM_2.5_, such as using a toaster or microwave oven in homes with a gas stove to reduce NO_2_ concentrations [59], as well as replacing gas stoves with electric stoves [58]. Use of indoor air purification may also be a cost-effective way to effectively decrease exposure to NO_2_ and PM_2.5_ from both indoor and outdoor sources [82]. When designing home interventions, it is crucial to evaluate the size of a residence as it is an important component [83].

### 4.5. Limitations

We used the outdoor air quality data from the EPA from a monitoring station in Chelmsford, MA, rather than direct onsite measurement to represent the outdoor concentrations of NO_2_ and PM_2.5_ at the participant’s homes. This extrapolation did not account for the surrounding environment of the households, such as major roads or green space, that could influence the levels of outdoor air pollutants, as observed in other studies [24]. This may have affected the relationship between indoor and outdoor concentrations of NO_2_ and PM_2.5_. Determining the factors that explain the variability in indoor NO_2_ and PM_2.5_ would be easier if NO_2_ and PM_2.5_ sampling were performed only while cooking. A standard measure of ventilation was not conducted during the study, and collecting data on ventilation such as measuring indoor CO_2_ concentration, the number and timing of open windows, air conditioning use, and range ventilation hood use and effectiveness would be advisable in future sampling studies. We did not collect data on the home size and design, which could be a potential limitation in understanding the dispersion of NO_2_ and PM_2.5_, as home size has been observed to influence the concentration of NO_2_ and PM_2.5_ [80,84]. In addition, we observed that high-frequency air freshener use contributed to indoor PM_2.5_, but recording and evaluating different air freshener types would have enabled a better understanding of their relationship to indoor PM_2.5_ concentrations.

## 5. Conclusions

This study has identified factors that influence indoor NO_2_ and PM_2.5_ concentrations. In our cohort of low-income senior housing with gas stoves, indoor NO_2_ depended predominantly on the season and type of gas stoves used in these homes. Stoves with pilot lights increased indoor NO_2_ concentrations. Higher indoor PM_2.5_ depended predominantly on the season and use of air fresheners 6–7 days a week. We did not observe the percentage of time the stove was used was a significant predictor of indoor NO_2_ and PM_2.5_. Our findings imply that indoor NO_2_ and PM_2.5_ depend on many factors other than the outdoor levels [64]. Future studies should focus on quantifying building characteristics and ventilation capacity, sampling a broader range of home types, and taking more precise measurements of outdoor pollutant concentrations. An improved understanding of the factors affecting indoor NO_2_ and PM_2.5_ concentration levels can lead to the development of an efficient management strategy to control health risks from exposure to indoor NO_2_ and PM_2.5_.

## Figures and Tables

**Table 1:** Apartment characteristics.

Variables	Categories	*n* = 73	%
Season of environmental sampling in homes	Winter	14	19
	Spring	32	44
	Summer	18	25
	Fall	9	12
What type of housing is this?	Townhouse	20	27
	Apartment Building	53	73
Kitchen Characteristics			
How do you light the stove burners?	Autoignition	68	93.15
	Pilot light	5	6.85
Is there a working hood or vent present?	Yes (recirculating vent)	34	46.58
	No	39	53.42
The Kitchen layout is:	A room with entire-height walls separate from the living area	43	58.90
	A room with a wall open to the living area	30	41.10
Does tobacco smoke enter your apartment from other apartments or outside?	Yes	37	50.68
	No	34	46.58
	Missing	2	2.74
Does your vacuum cleaner have a HEPA filter?	Yes	13	17.81
	No	58	79.45
	Missing	2	2.74
How often is air freshener used in your house?	<6 days/week	49	67.12
	6–7 days/week	22	30.14
	Missing	2	2.74
Wall-to-wall carpeting in the living area?	Yes	32	43.84
	No	39	53.42
	Missing	2	2.74
Area rug in living space?	Yes	17	23.29
	No	53	72.60
	Missing	3	4.11
Do trucks typically drive on your street?	Yes, often	35	47.95
	Yes, occasionally	23	31.51
	No	11	15.07
	Missing	4	5.48
Are any of these close to your apartment?	Dry cleaner	55	75.34
	Gas Station	68	93.15
	Restaurant	68	93.15
	Bakery	57	78.08
	Auto body shop	57	78.08
	Truck loading/unloading area	63	86.30
	City bus stop	70	95.89

**Table 2:** Comparison of indoor and outdoor levels of NO_2_ and PM_2.5_ for the sampling period (5–7 days).

	*n*	Mean (SD)	Range		
Average indoor temperature (°C)	73	24.0 (2.3)	18.1–32.0		
Average indoor humidity (g/m^3^)	73	43.6 (12.0)	22.8–66.7		
Fraction of stove use	72	0.3 (0.2)	0–0.8		
	*n*	GM (GSD)	Range	Mean (SD)	*p*-value
NO_2_ (ppb)					
Indoor	73	21.8 (2.1)	4.0–148.0	28.6 (24.5)	
Outdoor	69	10.9 (1.7)	2.6–27.7	12.4 (5.9)	
Paired *t*-test *	69				<0.0001
PM_2.5_ (µg/m^3^)					
Indoor	73	16.2 (2.7)	1.8–305.4	30.4 (48.2)	
Outdoor	71	5.9 (1.8)	2.0–59.0	7.3 (7.6)	
Paired *t*-test *	71				<0.0001

SD = Standard Deviation; GM = Geometric Mean; GSD = Geometric Standard Deviation. Paired *t*-test was conducted on natural log-transformed outdoor and indoor NO_2_ and PM_2.5_ during the sampling period. A significance of *p* < 0.05 is denoted as *.

**Table 3:** Single-determinant models of indoor ln NO_2_ concentrations.

			95% CI		
Exposure Determinant	*n*	Estimate	Lower	Upper	*p*-Value
Stove Use	72	0.17	−0.57	0.92	0.65
Outdoor NO_2_	69	0.16	−0.16	0.48	0.33
Season (ref = winter)	73				
Fall *		−0.57	−1.13	−0.003	0.05
Spring *		−0.42	−0.85	0.0002	0.05
Summer *		−0.83	−1.30	−0.36	0.001
Stove Type (ref Autoignition) *	73	0.68	−0.04	1.33	0.04
Average Temperature (°C)	73	−0.02	−0.10	0.05	0.58
Average Humidity	73	−0.01	−0.02	0.004	0.16
Vent (ref—yes) No	73	−0.08	−0.42	0.25	0.62
Room Type (ref—Wall separates kitchen and living area)	73	−0.11	−0.45	0.23	0.53
Outside Tobacco Smoke (ref—No) Yes	71	0.05	−0.29	0.39	0.78
Vacuum with HEPA Filter (ref—yes) No	73	−0.16	−0.61	0.28	0.47
Air Freshener Use (ref ≤ 6 day/weeks) 6–7 days/week	71	−0.25	−0.61	0.12	0.18
Trucks Nearby (ref—No) Yes	69	0.33	−0.13	0.78	0.16
Gas Station Nearby (ref—No) Yes	73	−0.43	−1.08	0.23	0.20

A significance of *p* < 0.05 is denoted as *.

**Table 4:** Single-determinant models of indoor ln PM_2.5_ concentrations.

			95% CI		
Exposure Determinant	*n*	Estimate	Lower	Upper	*p*-Value
Stove Use	72	−0.20	−1.25	0.86	0.71
Outdoor PM_2.5_	71	−0.14	−0.55	0.27	0.51
Season (ref = winter) Fall	73				
Fall		−0.27	−1.08	0.55	0.52
Spring		−0.51	−1.13	0.10	0.10
Summer *		−0.74	−1.43	−0.06	0.03
Stove Type (ref Autoignition)	73	−0.48	−1.39	0.43	0.30
Average Temperature (°C)	73	−0.10	−0.20	0.003	0.06
Average Humidity	73	−0.02	−0.03	0.003	0.11
Vent (ref yes)	73	−0.34	−0.79	0.12	0.15
Room Type (ref Wall separates kitchen and living area)	73	0.09	−0.38	0.56	0.70
Outside Tobacco Smoke (ref No) Yes	71	−0.04	−0.51	0.43	0.86
Vacuum with HEPA filter (ref Yes) No	71	0.46	−0.15	1.06	0.14
Wall-to-Wall Carpet (ref No) Yes	71	0.03	−0.44	0.51	0.89
Air Freshener Use (ref ≤ 6 days/week) 6–7 days/week *	71	0.77	0.29	1.25	0.0016
Trucks Nearby (ref—No) Yes	69	−0.12	−0.78	0.54	0.73
Gas Station Nearby (ref—No) Yes *	73	1.13	0.25	2.01	0.01

A significance of *p* < 0.05 is denoted as *.

**Table 5:** Multiple regression model of indoor ln NO_2_ concentrations (*n* = 72).

			95% CI		
Exposure Determinant	*n*	Estimate	Lower	Upper	*p*-Value
Stove Use Fraction	72	0.24	−0.41	0.90	0.47
Season (ref = winter) Fall *		−0.67	−1.19	−0.16	0.01
Spring *		−0.51	−0.89	−0.12	0.01
Summer *		−0.99	−1.43	−0.55	<0.0001
Stove Type (ref = autoignition) Pilot Light *		1.04	0.46	1.62	0.0004

A significance of *p* < 0.05 is denoted as *.

**Table 6:** Multiple regression model of indoor ln PM_2.5_ concentrations (*n* = 70).

			95% CI		
Exposure Determinant	*n*	Estimate	Lower	Upper	*p*-Value
Stove Use Fraction	70	−0.15	−1.14	0.84	0.76
Season (ref = winter) Fall		−0.31	−1.08	0.46	0.43
Spring *		−0.58	−1.16	0.01	0.05
Summer *		−0.69	−1.35	−0.04	0.04
Air Freshener Use (ref = never) 6–7 days/week *		0.79	0.32	1.26	0.001

A significance of *p* < 0.05 is denoted as *.

## Data Availability

The datasets used and analyzed during the current study are available from the corresponding author upon reasonable request.
